# Concurrent phenylketonuria and immune-mediated central nervous system involvement: a case report highlighting rare co-occurrence

**DOI:** 10.1186/s12883-025-04613-7

**Published:** 2026-01-09

**Authors:** Junjie Zhao, Hongjing Yan, Zhixuan Li, Xiaoxiao Wang, Yining Wang, Xiaoyu Zhang

**Affiliations:** 1 Department of Neurology, Handan First Hospital, Handan City, China; 2 Electromyography Laboratory, Handan Central Hospital, Handan City, China

**Keywords:** Phenylketonuria, Immune-mediated central nervous system involvement, Spinal cord, Magnetic resonance imaging, Oligoclonal bands

## Abstract

**Background:**

White-matter abnormalities are common in phenylketonuria (PKU) and are typically attributed to metabolic hypomyelination or intramyelinic edema. Immune-mediated CNS involvement co-occurring with PKU has rarely been described in adults.

**Case:**

An 18-year-old male university freshman diagnosed with classical PKU presented with a 3-week history characterized by progressive bilateral lower-limb weakness and gait instability. Cervical spinal magnetic resonance imaging (MRI) revealed intramedullary lesions, indicating possible immune-mediated CNS involvement overlapping PKU. Cerebrospinal fluid (CSF) analysis was acellular but demonstrated positive oligoclonal IgG bands. Notable clinical and radiological improvement was observed following the administration of high-dose intravenous methylprednisolone.

**Diagnosis:**

Considering the acute course, brain cord involvement, positive OCB, and steroid responsiveness, an acute immune-mediated CNS lesion overlapping PKU was diagnosed. The symmetric diffusion‑restricted brain lesions raised the possibility of concomitant PKU‑related intramyelinic edema.

**Treatment:**

High‑dose intravenous methylprednisolone (1 g/day for 3 days) followed by an oral taper was administered, together with strict low‑phenylalanine diet and vitamin supplementation.

**Outcome:**

Cognitive slowing and lower‑limb weakness improved substantially within 3 weeks. One‑month follow‑up MRI showed regression of spinal lesions and slight reduction of supratentorial white‑matter abnormalities.

**Conclusions:**

This case highlights a potential metabolic–immune overlap in PKU. Positive OCB and steroid‑responsive cord lesions support an inflammatory component, while symmetric diffusion‑restricted brain lesions suggest concurrent metabolic vulnerability. Longitudinal imaging and metabolic control (serial plasma phenylalanine/tyrosine, myelin‑sensitive MRI metrics) are essential to refine diagnosis and guide the need for disease‑modifying therapy.

## Introduction

Phenylketonuria (PKU) is an infrequent autosomal recessive metabolic disorder resulting from pathogenic variants in the phenylalanine hydroxylase (PAH) gene. A deficiency in PAH activity leads to a systemic accumulation of phenylalanine and its metabolites. Although dietary management is typically established during infancy, cessation of a low-phenylalanine diet post-puberty may still trigger progressive cognitive decline, motor impairments, and various systemic complications in adulthood [1].

Central nervous system (CNS) inflammatory demyelinating diseases, such as multiple sclerosis (MS), acute disseminated encephalomyelitis (ADEM), and neuromyelitis optica spectrum disorders, represent a group of disorders characterized by inflammation and demyelination of the CNS [2]. These conditions share common pathophysiological mechanisms, including immune-mediated damage to myelin and axons, which lead to neurological deficits. The diagnostic process involves assessing the dissemination of lesions over time and space, identifying CSF-restricted oligoclonal IgG bands, and evaluating the response to immunomodulatory therapy. To the best of our knowledge, no comprehensive study has systematically investigated the coexistence of PKU with immune-mediated CNS involvement; only isolated reports suggest a potential link between PKU and immune dysregulation [3].

This case report is the first to document acute inflammatory demyelinating disease in an adult with classic PKU, underscoring the need for further investigation.

## Case report

**Fig. 1:**
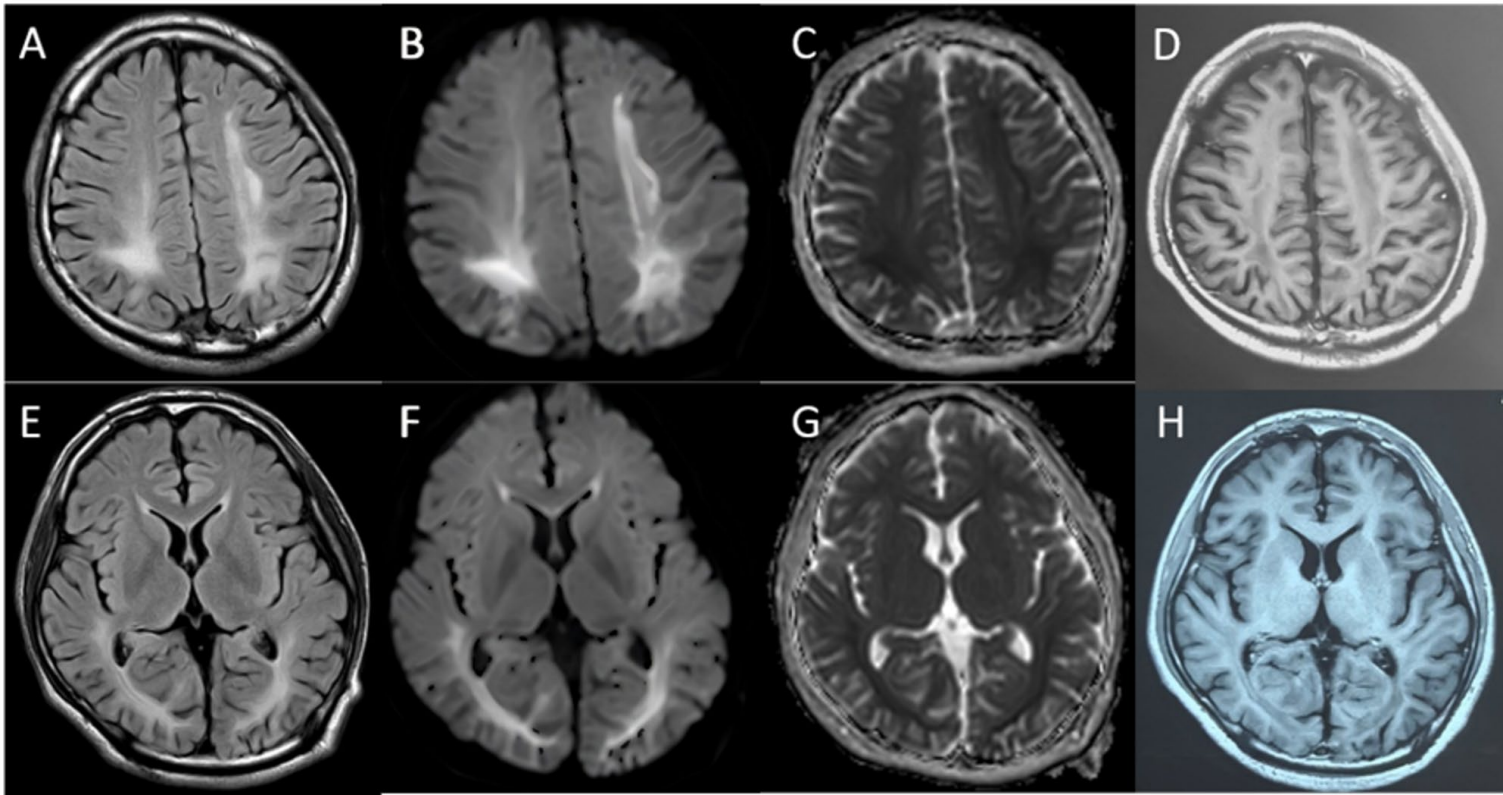
Axial FLAIR (**A**, **E**) MRI shows increased signal intensity in the frontal, parietal and occipital white matter. Matching axial plane DWI (**B**, **F**) and ADC (**C**, **G**) maps show restricted diffusion in the corresponding areas. Contrast-enhanced (**D**, **H**) showed no significant enhancement

**Fig. 2:**
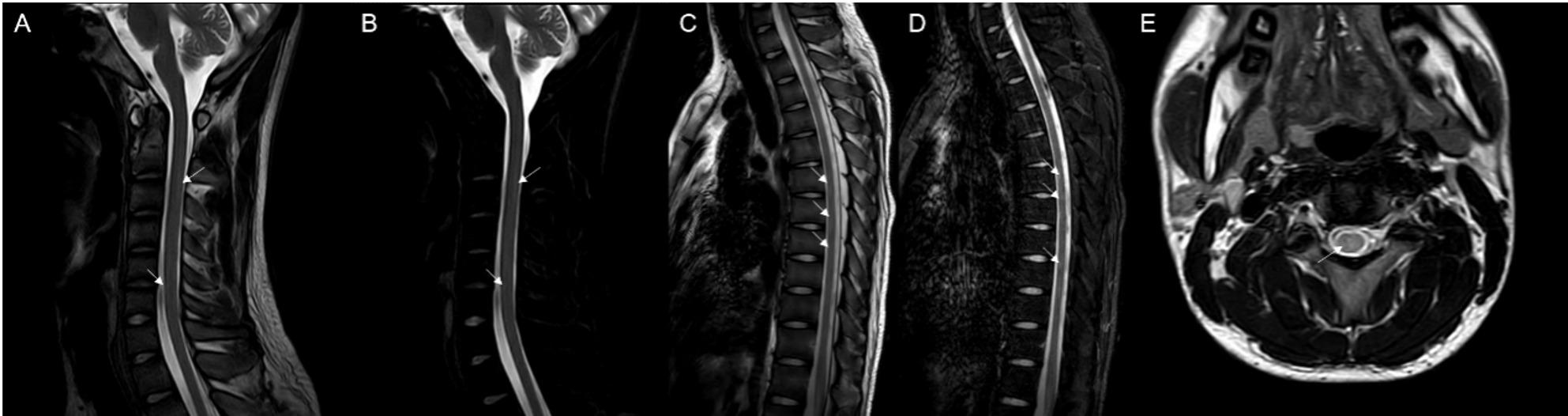
Cervical and thoracic spine MRI before treatment: At the C2–3 vertebral level, a stripe-like hyperintensity is seen on both T2WI (**A**) and fat-suppressed T2WI (**B**) along the dorsal aspect of the spinal cord, with ill-defined borders. At C5–6, a similar subtle stripe-like hyperintensity is noted along the ventral cord, also poorly marginated. Multiple small patchy hyperintensities on T2WI (**C**) and fat-suppressed T2WI (**D**) are scattered along the ventral cord from T6 to T9, all with indistinct borders. At the C2–3 vertebral level, axial images (**E**) show a slightly hyperintense stripe along the dorsal aspect of the spinal cord with ill-defined borders

**Fig. 3:**
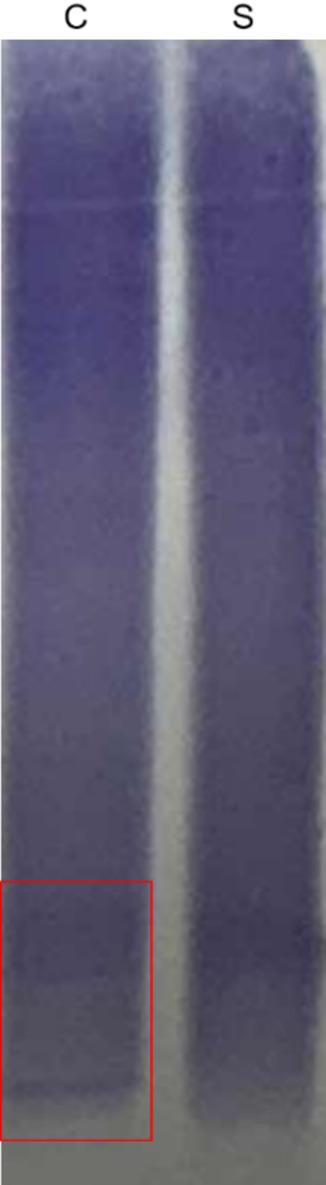
Isoelectric focusing (IEF) lectrophoresis pattern. C: cerebrospinal fluid, S: serum

**Fig. 4:**
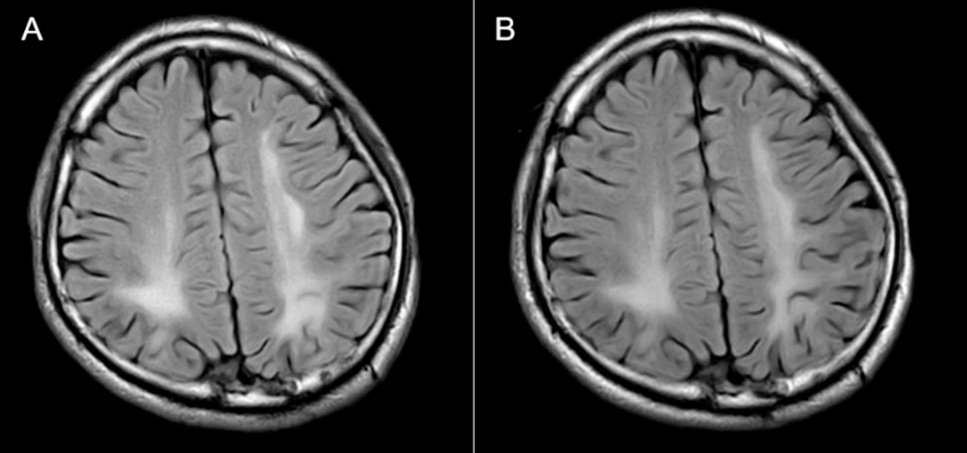
Post-treatment axial FLAIR (**B**) demonstrated persistence of the symmetrical supratentorial white-matter hyperintensities, with a slightly reduced extent compared with the pre-treatment axial FLAIR (**A**)

**Fig. 5:**
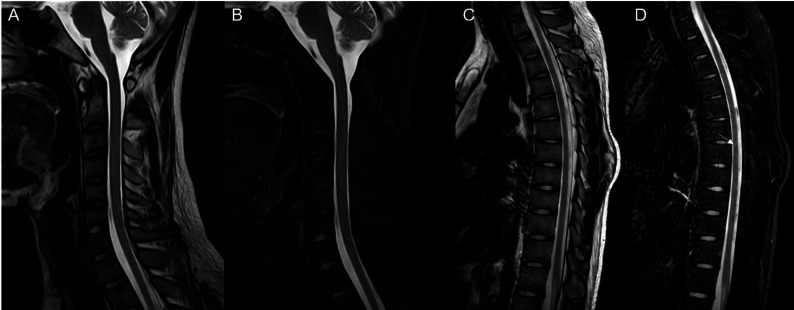
Cervical and thoracic spine MRI after treatment: Fat-suppressed images (**D**) demonstrate that the lesion at the T6–7 level has markedly decreased in extent, with no definite abnormal signal now visible at any previously affected segment (**A**, **B**, **C**)

We present the case of an 18-year-old male university freshman who was admitted to our department on 14 July 2025, with a 20-day history of progressive bilateral lower-limb weakness. The patient was diagnosed with classical PKU by newborn screening in the first week of life and immediately started on a phenylalanine-restricted diet. This regimen, along with unspecified oral medications, was continued until age 6, when all treatment was discontinued. Developmental milestones were normal except for mild lifelong dysarthria. For approximately six years he has experienced intermittent bilateral hand tremor that is postural, most noticeable under stress, first appeared after dietary discontinuation, and improves when the low-phenylalanine diet is reinstated; the consulting neurologist attributes it to chronic hyperphenylalaninemia rather than to drugs or essential tremor. The family history was unremarkable, with both parents and two elder sisters reported to be in good health. Three weeks prior to admission, the patient began experiencing difficulty in lifting both legs while walking, characterized by weakness during hip flexion and knee extension. Despite these symptoms, he remained ambulatory and did not experience any falls. The patient exhibited persistent but non-progressive weakness, without associated numbness, pain, sphincter disturbances, back pain, or involvement of the upper limbs. Over the preceding five months, the patient experienced an unintentional weight loss of 5 kg, alongside a reduced appetite and poor sleep quality. Upon admission, the vital signs recorded were as follows: pulse rate at 85 beats per minute and blood pressure at 132/91 mmHg; temperature and respiratory rate were not documented. General examination revealed fair skin and light blond hair. The patient was alert but demonstrated psychomotor slowing and difficulty with word-finding, although orientation and memory remained intact. Ocular motility was complete, but subtle horizontal nystagmus was observed during left lateral gaze. The remainder of the cranial nerve examination was unremarkable. Muscle strength was assessed as 5/5 in the upper limbs and 4/5 in the lower limbs, with normal muscle tone. Sensory examination, deep sensation, and coordination tests yielded no significant findings. The patient’s gait was characterized by bilateral foot drop and reduced step height, with a negative Romberg sign. Deep tendon reflexes were brisk (+++) and symmetrical. Hoffmann’s sign was positive on the left side; Babinski’s sign was positive on the right and equivocal on the left; Chaddock’s sign was positive bilaterally. The examination revealed an absence of meningeal signs. Comprehensive laboratory evaluations, including a complete blood count, coagulation profile, assessments of liver and renal function, electrolyte levels, vitamin B12 levels, infectious panels, antinuclear antibody (ANA) profile, thyroid function tests, erythrocyte sedimentation rate (ESR), and routine urinalysis, were all within normal parameters. However, sCSerum homocysteine levels were significantly elevated at 95.8 µmol/L, compared to the reference range of 0–15 µmol/L. MRI of the brain indicated extensive, symmetrical, patchy T2/FLAIR hyperintensities within the periventricular and deep white matter of the frontal, parietal, temporal, and occipital lobes (refer to Fig. 1). Cervical spinal MRI revealed intramedullary T2 hyperintensity (refer to Fig. 2), while thoracic imaging showed equivocal signal changes. The initial differential diagnoses included: (1) PKU, (2) intramedullary signal abnormality suggestive of immune-mediated CNS involvement, and (3) hyperhomocysteinemia. A therapeutic regimen comprising a low-phenylalanine diet, along with folate and vitamin B12 supplementation, was initiated. To rule out immune-mediated CNS demyelination, a lumbar puncture was performed. The opening CSF pressure was measured at 100 mmH₂O. CSF analysis indicated normal cell count and chemistry, although CSF creatine kinase levels were slightly elevated at 47.8 U/L. Oligoclonal IgG bands were present, exhibiting pattern 2 (refer to Fig. 3), and the 24-hour intrathecal IgG synthesis rate was calculated at 4.61 mg/dL (reference range: −9.9 to 3.3 mg/dL). All serum and CSF autoantibodies associated with CNS demyelination tested negative via Cytometric Bead Array. On July 25, 2025, the patient was transferred to the Department of Neurology at Peking University People’s Hospital. Further metabolic evaluation revealed significantly elevated levels of urinary phenylpyruvate (218.94 µmol/mmol Cr; reference range: 0–0.10) and phenyllactate (37.44 µmol/mmol Cr; reference range: 0–8.84). Serum homocysteine levels remained elevated at 46.7 µmol/L, while folate levels exceeded 20 ng/mL (reference range: 4.2–19.8 ng/mL). ceruloplasmin was within normal limits. Contrast-enhanced MRI of the brain showed no significant enhancement (refer to Fig. 1), and chest computed tomography (CT) was unremarkable. Multimodal evoked potentials indicated bilateral suboptimal waveforms: brainstem auditory evoked responses exhibited prolonged latencies and reduced amplitudes on the right side; visual evoked potentials showed delayed P100 latency on the right with reduced amplitudes bilaterally; and lower-limb somatosensory evoked potentials demonstrated significantly attenuated cortical responses. The final diagnoses were: (1) immune-mediated CNS demyelinating disease, and (2) PKU. The patient received intravenous methylprednisolone at a dosage of 1 g daily for three days, followed by oral prednisone at 60 mg per day with a tapering schedule of 15 mg per week. The patient was discharged on hospital day 18 (1 August 2025). At the outpatient follow-up on 18 August 2025, there was an almost complete resolution of cognitive slowing, word-finding difficulty, and lower-limb weakness, with a return to normal gait. No adverse effects were observed during hospitalization or follow-up. A repeat brain MRI on post-discharge day 26 (27 August 2025) revealed persistent, albeit slightly reduced, symmetrical supratentorial white matter hyperintensities (refer to Fig. 4), while cervical and thoracic spinal MRI results were unremarkable (refer to Fig. 5).

## Discussion

An 18-year-old with classic PKU rapidly developed bilateral leg weakness. Cervicothoracic-cord MRI showed focal T2-hyperintense lesions, CSF revealed pattern-2 oligoclonal bands, and high-dose methylprednisolone induced prompt lesion regression. These findings suggest an acute immune-mediated CNS injury superimposed on metabolically fragile white matter. To date, clinical cases associating PKU with immune-mediated injury are exceedingly rare; only Beke et al. [3] have reported a 3.5-year-old child with PKU who developed pyogenic sacroiliitis and a left iliopsoas abscess, suggesting a potential link between PKU and immune dysregulation. Such cases are exceedingly rare in the literature and hold significant clinical and scholarly importance. This single case only cautiously suggests that, beyond its well-established metabolic profile, PKU may occasionally overlap with immune-mediated processes. Given the rarity of such associations, the most plausible interpretation is simple co-occurrence. Whether any underlying “metabolic–immune” interaction exists remains unsupported by current evidence and awaits larger-scale studies for clarification. Villasana et al. (1989) were the first to document, through the use of MRI and CSF neurotransmitter metabolites, that in adults with PKU who experience neurological decline, the cessation of a low-phenylalanine diet post-puberty can lead to progressive spasticity in adulthood, even if dietary management was adequate during infancy. In their index case, the patient discontinued dietary restrictions at age 12, developed spastic paraparesis at 28, and required two months of resumed low-phenylalanine intake before motor function improved [1]. In contrast, our patient demonstrated significant clinical recovery within approximately 20 days of reinitiating the low-phenylalanine diet in conjunction with high-dose glucocorticoid pulse therapy. This suggests that the rapid improvement was more likely due to the swift response of immune-mediated CNS involvement to corticosteroids.

Phenylalanine hydroxylase (PAH) deficiency results in systemic hyperphenylalaninemia; however, the effects of elevated phenylalanine levels are tissue-specific. Despite the high expression of PAH in both the liver and kidney [4, 5], PAH deficiency does not lead to overt structural damage in these organs. In the absence of treatment, the primary clinical manifestations of PKU are observed in the brain [6, 7]. Multi-tissue metabolomic analyses further reveal disrupted energy metabolism and increased oxidative stress within the PKU brain [8], while no similar abnormalities are observed in the liver, the organ primarily affected by PAH deficiency. This highlights the “brain-specific” pathogenesis of PKU. These neurological alterations are generally interpreted as hypomyelination or myelin dysplasia, rather than classic immune-mediated demyelination [9]. Advanced imaging techniques, such as myelin water fraction mapping and magnetic resonance spectroscopy, have demonstrated microstructural changes even in macroscopically normal white matter, supporting the concept of “developmental/metabolic myelin vulnerability” in PKU [10, 11].

Even among patients with classic PKU who receive early treatment, white-matter lesions are remarkably prevalent. A comprehensive analysis of over 300 neuroimaging and neuropsychological studies on PKU revealed a cumulative prevalence of 93% (290 out of 312 cases) [9]. These lesions are typically bilateral, symmetrical, and confluent, initially manifesting in the deep periventricular white matter and primarily affecting the occipital and parietal lobes, before progressing to the frontal and temporal lobes and the subcortical arcuate fibers. In severe instances, the lesions may extend to the splenium of the corpus callosum, the posterior limb of the internal capsule, and, in some cases, the brainstem and cerebellum. Infratentorial structures, such as the brainstem and cerebellum, are infrequently affected and generally only in severe or chronically poorly managed cases, with the extent of involvement positively correlating with supratentorial disease [12]. The cerebral white-matter abnormalities observed in our patient align with these PKU-associated patterns. To date, involvement of the spinal cord in PKU has been scarcely documented.

Mechanistic investigations into white-matter lesions in PKU have consistently demonstrated, through both imaging and electrophysiological studies, that these alterations result from chronic intramyelinic edema rather than demyelination [11, 13, 14]. Hyperphenylalaninemia competitively inhibits the transport of aromatic amino acids, thereby disrupting normal lipid synthesis in oligodendrocytes and leading to intramyelinic edema, characterized by the retention of water between myelin lamellae with vacuole formation, without actual loss of myelin or axons. This process is metabolically dependent and reversible, as evidenced by significant improvements in MRI abnormalities following adherence to a strict low-phenylalanine diet for a duration of two months or more. In the case of our patient, lesions in the cervical and thoracic spinal cord were observed on routine MRI and showed regression within one month following steroid pulse therapy, suggesting an active immune-mediated demyelinating process rather than the typical metabolic intramyelinic edema associated with PKU. Research on CSF in PKU has predominantly concentrated on metabolites and neurotransmitters [15–19], DNA damage [20, 21], energy depletion [22], and oxidative stress, as well as blood–brain barrier permeability to amino acids [23, 24]. Systematic investigations of neuro-inflammatory markers such as intrathecal IgG synthesis and oligoclonal bands (OCB) are scarce. In one reported adult with confirmed PKU, CSF IgG oligoclonal bands were negative—a finding used to distinguish PKU from CNS disorders such as MS that are characterized by intrathecal IgG production [25].

The patient in question exhibits several criteria indicative of an inflammatory demyelinating process, as opposed to white-matter changes solely associated with PKU. These criteria include: (1) an acute onset with rapid progression; (2) concurrent involvement of both the brain and cervical spinal cord; (3) the presence of positive CSF oligoclonal bands, suggesting intrathecal immunoglobulin synthesis; and (4) a significant response to glucocorticoid pulse therapy. Collectively, these clinical features align more closely with an inflammatory demyelinating event within the MS spectrum or a clinically isolated syndrome (CIS), rather than the chronic, symmetrical, and relatively indolent white-matter abnormalities characteristic of PKU-associated hypomyelination. However, it is important to note that ADEM can also result in transient oligoclonal bands. Consequently, a definitive classification necessitates evidence of dissemination in time and space, a history of relapses, and serial imaging studies. Given the broad phenotypic spectrum of pediatric and adolescent inflammatory demyelinating disorders, maintaining an open differential diagnosis and conducting systematic follow-up are crucial [26]. This case highlights the importance of evaluating adults with PKU who present with acute spinal or cerebral symptoms should be evaluated promptly for an overlapping immune-mediated event, including CSF analysis and consideration of immunotherapy.

We propose an integrated “susceptibility–trigger–manifestation” framework, wherein chronic hyperphenylalaninemia initially induces a metabolically driven, structurally fragile myelin state in individuals with PKU. Upon the occurrence of additional immune or environmental triggers, such as infection or physiological stress, this metabolically primed state may exacerbate the neuro-inflammatory response, potentially leading to an acute inflammatory demyelinating event. This model is consistent with preclinical findings indicating that elevated phenylalanine levels can activate microglia and cause myelin damage [27]. However, systematic clinical evidence remains limited, necessitating further case series and mechanistic studies to substantiate this hypothesis.

The patient’s favorable response to glucocorticoids underscores the efficacy of standard empirical therapy for immune-mediated involvement [28]. Simultaneously, it is imperative to pursue aggressive metabolic optimization, which includes stringent control of blood phenylalanine levels, assessment of tetrahydrobiopterin (BH4) responsiveness, and consideration of large neutral amino acid (LNAA) supplementation or other innovative phenylalanine-lowering strategies to mitigate relapse risk and enhance myelin plasticity [29]. The initiation of disease-modifying therapy (DMT) should be tailored to the individual, informed by the robustness of MS diagnostic criteria (dissemination in time and space, history of relapses, imaging activity), the estimated risk of relapse, the safety profile of the therapy, and the presence of any underlying metabolic disorders.

This report is subject to several limitations. The lack of histopathological data hinders the precise differentiation between the contributions of underlying hypomyelination and pure inflammatory demyelination. Additionally, the brief follow-up period limits the ability to definitively classify the condition within the MS spectrum. In addition, serial plasma phenylalanine levels were not monitored, PAH genotyping was unavailable to explain phenotypic variability, CSF myelin basic protein was not measured to confirm active demyelination, and contrast-enhanced spinal imaging was not performed. We did not test for severe homocysteine-metabolism defects such as MTHFR deficiency. We recommend longitudinal monitoring employing quantitative MRI metrics, such as myelin water fraction and diffusion tensor imaging parameters, alongside fluid biomarkers like neurofilament light chain, to dynamically evaluate myelin and axonal integrity. Establishing a registry of inflammatory demyelinating events within the PKU population would aid in elucidating causality and clinical heterogeneity [11].

## Conclusion

This case emphasizes the rare co-occurrence of PKU and CNS demyelinating disease, highlighting the lack of CSF oligoclonal-band data in PKU patients and the need for further investigation. Acute immune-mediated CNS involvement can occur in PKU, with features similar to MS. Although PKU-related white-matter changes and an acute inflammatory demyelinating event were observed in the same patient, this co-occurrence does not necessarily imply a mechanistic link; an equally plausible view is that the two processes simply coexist without directly influencing each other. Clinically, we suggest starting immunotherapy quickly alongside strict metabolic management, with regular imaging and biomarker monitoring to improve diagnosis, assess relapse risk, and refine prognosis.

## Data Availability

All relevant data are included in the article. All relevant data are presented within the article and its figures. Additional data may be requested from the corresponding author without breaching patient confidentiality.

## Abbreviations

PKU: phenylketonuria
CSF: cerebrospinal fluid
MRI: magnetic resonance imaging
CNS: central nervous system
